# The impact of surgical volume on outcomes in newly diagnosed colorectal cancer patients receiving definitive surgeries

**DOI:** 10.1038/s41598-024-55959-w

**Published:** 2024-04-08

**Authors:** Chiu-Mei Yeh, Tzu-Yu Lai, Yu-Wen Hu, Chung-Jen Teng, Nicole Huang, Chia-Jen Liu

**Affiliations:** 1 Division of Transfusion Medicine, Department of Medicine, Taipei Veterans General Hospital, No. 201 Shipai Road, Sec. 2, Taipei, 11217 Taiwan; 2 Institute of Public Health, National Yang Ming Chiao Tung University, Taipei, Taiwan; 3 Division of Radiation Oncology, Department of Oncology, Taipei Veterans General Hospital, Taipei, Taiwan; 4 School of Medicine, National Yang-Ming University, Taipei, Taiwan; 5 Division of Hematology and Oncology, Department of Medicine, Far Eastern Memorial Hospital, New Taipei City, Taiwan; 6 Institute of Hospital and Health Care Administration, National Yang Ming Chiao Tung University, No. 155 Linong St., SeCc. 2, Beitou District, Taipei, 11217 Taiwan; 7 Institute of Emergency and Critical Care Medicine, National Yang Ming Chiao Tung University, Hsinchu, Taiwan

**Keywords:** Cancer epidemiology, Risk factors

## Abstract

Colorectal cancer (CRC) patients who receive cancer surgeries from higher-volume providers may have better outcomes. However, the definitions of surgical volume may affect the results. We aim to analyze the effects of different definitions of surgical volume on patient outcomes. We conducted a nationwide population-based study in Taiwan that enrolled all patients who underwent definitive surgery for newly diagnosed CRC. We used three common definitions of surgical volume: total volume means the total surgical number conducted by the same provider during the study period; cumulative volume was calculated as the number of operations the surgeon performed before the index procedure; annual volume was calculated as the number of times the surgeon had been responsible for surgery during the index year. In this study, we included 100,009 newly diagnosed CRC patients, including 55.8% males, of median age 66 years at diagnosis (range 20–105 years). After adjustment for the patient and provider characteristics, we found that CRC patients receiving definitive surgery by higher-volume providers had better outcomes, especially where surgeon volume may play a more important role than hospital volume. The cumulative volume could predict the 5-year mortality of the study cohort better than the total and annual volume.

## Introduction

Colorectal cancer (CRC) is one of the most common cancers worldwide. Surgery is the cornerstone of treatment for CRC. More than half of newly diagnosed CRC patients receive surgical resection of the primary tumor, according to the Taiwan Cancer Registry^1^. Radical CRC surgery requires an en-bloc removal of the primary tumor with the associated mesentery to ensure adequate surgical margins, which is the most important prognostic factor for local control and survival^2,3^. Both colectomy and total mesorectal excision are common surgical interventions that general surgeons or colorectal specialists can perform in different levels of hospitals. Much of the literature has demonstrated that higher provider volume (i.e., hospital or surgeon volume) is associated with better surgical outcomes and long-term survival^4–7^. Some may argue that provider volume is hardly a proxy for other critical characteristics of providers, such as surgical technique or decision-making of the individual surgeon. Fundamental infrastructures of the hospital, including the quality of clinical care, equipment of the intensive care unit, and multidisciplinary teamwork, are essential considerations. However, it serves as a simple and intuitive indicator for measurement^5,8^.

Since the association between surgical volume and mortality was first described in the 1970s^9^, many publications have confirmed a positive volume-outcome relationship. Both surgical and medical therapeutic volumes have been reported to correlate with patient outcomes. For example, Ross et al. wrote that admission to higher-volume hospitals would reduce the 30-day mortality for acute myocardial infarction, heart failure, and pneumonia^10^. Schrag et al. noted that hospital surgical volume was effective in predicting clinical outcomes in patients who underwent surgery for colon cancer^11^. Several studies in Taiwan have also discussed volume and outcome. Lin et al*.* reported physician volume predicting inpatient mortality among ICU patients with pneumonia^12^. However, not all empirical evidence supports the volume-outcome relationship. Leonard et al*.* showed a volume effect on circumferential resection margin, R0 resection rate, sphincter preservation rate, and the number of nodes examined after chemoradiotherapy in patients with stage II–III rectal cancer; however, no volume effect was observed for recurrence and overall survival rates^6^. Yasunaga et al*.* reported that neither hospital nor surgeon volume was associated with postoperative complications in patients after colectomy^13^.

Recent studies with more delicate methods by applying advanced statistical and analytical strategies have provided different findings. Some studies argue that volume is not adjusted for confounding variables. For instance, Chen et al*.* reported high surgeon and hospital surgical volume as a significant contributor to outcomes of patients with breast cancer^14^. In contrast, Kuo et al. reported no association between surgeon volume and breast cancer recurrence or survival after controlling for patient and provider characteristics using multilevel mixed-effect models^15^. Panageas et al*.* demonstrated that when implementing random-effect models or generalized estimating equations in volume-outcome studies to adjust for the clustering effect of surgeons, the strength of statistical significance attenuates. In contrast, conventional statistical methods often result in overly narrow confidence intervals and could lead to biased interpretations^16^.

In addition, the definitions of surgical volume also affected surgical outcomes. Several studies used different definitions of surgical volume and combinations^17–19^. However, no studies have been published to date comparing the effects of different definitions of surgical volume on patient outcomes. To resolve this issue, we used Taiwan’s National Health Insurance Research Database (NHIRD) to analyze the outcomes of patients who received definitive surgeries for CRC by using different definitions of surgical volume.

## Materials and methods

### Data sources

Taiwan’s National Health Insurance (NHI) program was established on March 1, 1995. It covers more than 99.9% of Taiwan's population, including outpatient, inpatient, emergency, dental, and traditional Chinese medical services, as well as surgical procedures and prescription medicine. The NHIRD provides nationwide population-based data, making it a valuable resource for population-based health research. To ensure patient confidentiality, data were retrieved and analyzed by on-site analysis at the Health and Wellness Data Science Center via remote connection to the Ministry of Health and Welfare server. All patients with severe diseases, of which cancer is included, are enrolled in the Registry for Catastrophic Illness Patients (RCIP) and receive copayment exemption under the NHI program. The NHIRD contains information on the demographic characteristics of hospitals and physicians, ambulatory care, admissions, procedures, diagnoses, and prescribed medications. The diagnosis coding system is used in line with the International Classification of Diseases (ICD) 9th revision system to classify diagnostic, health services utilization, and death data^20^.

All personally identifiable information in the NHIRD is encrypted to ensure privacy and confidentiality. The Bureau of National Health Insurance and the National Health Research Institutes have established regulations to safeguard the confidentiality of the data. This study has received approval from the Institutional Review Board at Taipei Veterans General Hospital, affirming its compliance with ethical research standards (2019-07-054BC). All study methods were performed per relevant guidelines and regulations of Taipei Veterans General Hospital in Taiwan. The Taipei Veterans General Hospital ethical committee waived the informed consent form.

### Study population

The patients enrolled in this study were newly diagnosed with CRC based on the ICD-9-CM major codes 153–154 and were to be registered in the RCIP from January 1, 2005 to December 31, 2016. Diagnosis of CRC requires pathological proof for enrollment in the RCIP. Those diagnosed with CRC before January 1, 2005 were not enrolled. CRC patients diagnosed under age 20 and those who did not identify sex and the essential information for providers were excluded.

### Surgical volume

**Figure 1:**
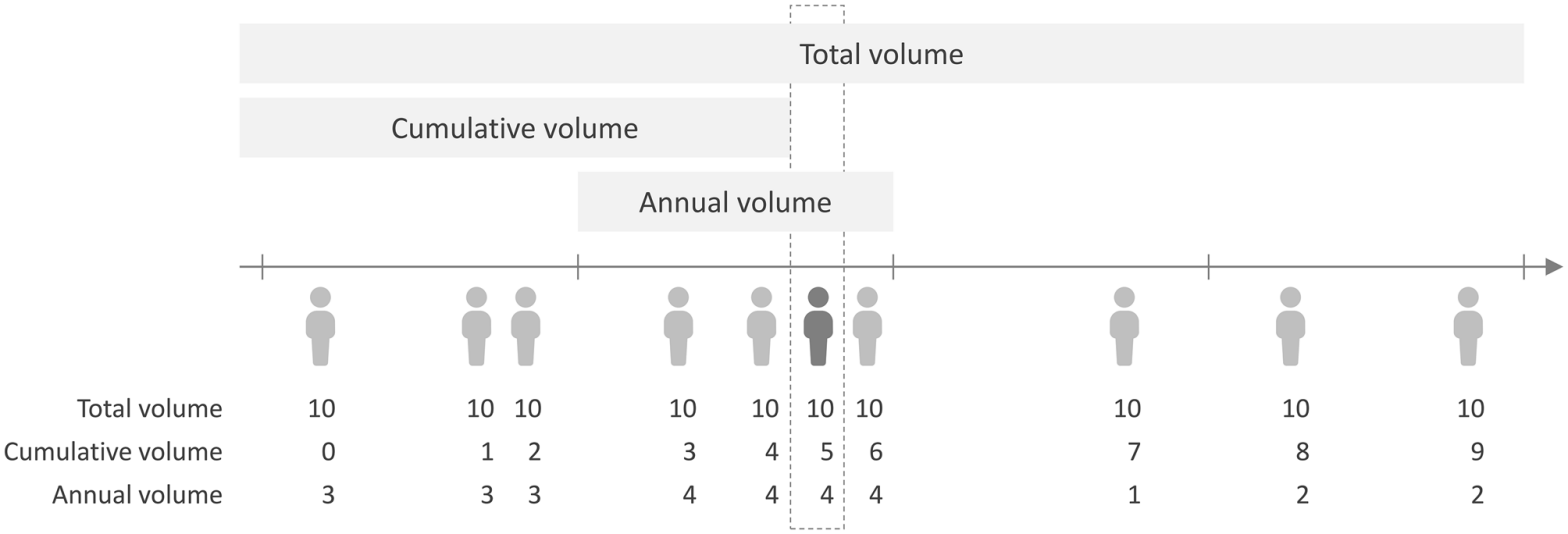
The definitions of surgical volume. This surgeon performed on 10 patients during the study period. The darker patient example shows the total, cumulative, and annual volume calculations.

The volume of CRC definitive surgeries by each surgeon and hospital was calculated. We used three common definitions of surgical volume. Total volume means the total surgical number conducted by the same provider during the study period. Cumulative volume was calculated as the number of operations the surgeon performed before the index procedure. In contrast, annual volume was calculated as the number of times the surgeon had been responsible for surgery during the index year. Figure 1 shows the number of CRC patients a surgeon surgically operated on at different times.

Notably, we divided all patients into quartiles based on the hospital and surgeon volumes, ranging from lowest to highest volume. All patients were stratified into four quartiles: lowest, middle-low, middle-high, and highest.

### Variables

The primary endpoint was death within 5 years from the first date of definitive surgery, which is a common indicator for the long-term outcome of cancer surgery^15,21–24^. Information on the date and cause of death is contained in the National Cause of Death Data. All patients enrolled in this study were followed until dropout from the NHI program, death from any cause, or the end of the year 2017.

Patient demographics include age, sex, comorbidities, urbanization, and insurance amount. Comorbidities, including hypertension, diabetes mellitus (DM), heart failure, chronic obstructive pulmonary disease (COPD), end-stage renal disease (ESRD), liver cirrhosis, and cerebrovascular accidents (CVA), were identified using ICD-9-CM codes from NHIRD. Patients’ socioeconomic status was categorized by degree of urbanization and monthly income. In addition, we used the physician and facility registries to construct hospital- and surgeon-level variables, including the hospital's level of accreditation, ownership, geographic region, as well as the surgeon's age, sex, and experience.

### Statistical analysis

CRC patients’ and providers’ demographic data were compared using Fisher’s exact test or chi-square test for categorical variables and the Mann–Whitney *U* test for continuous variables. The 5-year survival probability was measured using the Kaplan–Meier method from the time of diagnosis to death or last follow-up. The difference between groups was further estimated by a log-rank test. Univariate and multivariate Cox proportional hazards models were used to identify predictors of 5-year mortality among CRC patients. The surgeon-level random effects were adjusted using a frailty model for Cox regression in the multivariate analysis^12,25^. Furthermore, model discrimination was estimated using the Akaike information criterion (AIC) and the Bayesian information criterion (BIC), which showed how the different definitions of surgical volume affect the outcomes—the lower the AIC and BIC, the more explanatory the variables and the model are. Using the Harrell's C statistics calculation, we also conducted sensitivity analysis to determine the discriminatory ability of different definitions of hospital and surgeon volumes. Data management and all statistical analysis were performed using SAS 9.4 software (SAS Institute Inc., Cary, NC) and STATA statistical software, version 15.1 (StataCorp, College Station, TX). Statistical significance was defined as a *p *value of less than 0.05.

## Results

### Characteristics of the study population

**Figure 2:**
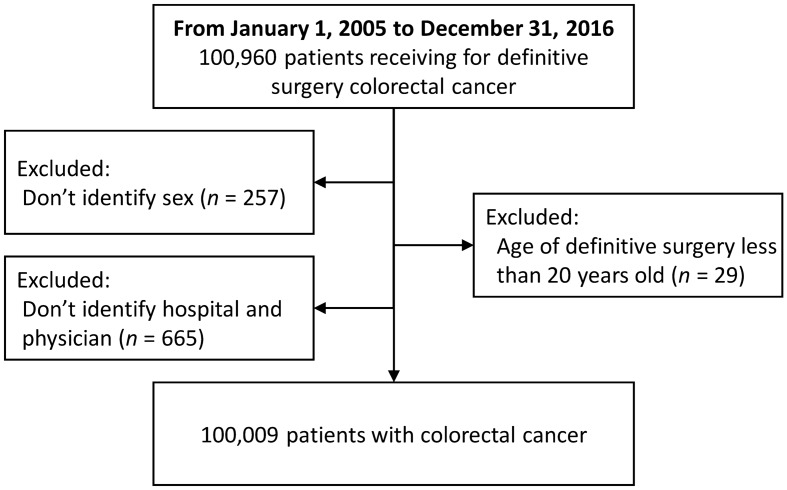
Patient selection flow chart.

We identified 100,960 newly diagnosed CRC patients who received definitive surgeries between January 1, 2005 and December 31, 2016. Of these, 257 patients did not reveal sex, 29 patients were under the age of 20, and 665 patients did not reveal the provider's basic information (Fig. 2). Therefore, the final cohort consisted of 100,009 patients, including 55,849 (55.8%) males and 44,160 (44.2%) females, of median age of 66 at diagnosis (range 20–105 years). The characteristics of CRC patients, hospitals, and surgeons are listed in Table 1. More than half of the surgeons were aged ≥ 45 years (57.4%), and the median experience of curative surgery was 8.6 (IQR 5.7–11.9) years. Patients were separated into four groups according to the surgeon’s total volume. The median total volume of CRC definitive surgeries was 575 (IQR 218–1,089) for surgeons. Patients aged ≥ 65, those who were male, those with comorbidities, patients having lower income, and those living in rural areas were more likely to undergo resection by lower-volume surgeons.

**Table 1:** Baseline characteristics of patients with colorectal cancer receiving definitive surgery.

Characteristics	Total *n* = 100,009	Total surgeon volume				*p* value
		Lowest quartile *n* = 24,699	Middle-low quartile *n* = 24,787	Middle-high quartile *n* = 25,320	Highest quartile *n* = 25,203	
Median age, years (range)	66 (20–105)	68 (20–101)	66 (20–103)	66 (20–105)	65 (20–101)	< 0.001
Age, years						
≥ 65	54,612 (54.6)	14,579 (59.0)	13,647 (55.1)	13,470 (53.2)	12,916 (51.2)	< 0.001
< 65	45,397 (45.4)	10,120 (41.0)	11,140 (44.9)	11,850 (46.8)	12,287 (48.8)	
Sex						
Male	55,849 (55.8)	14,111 (57.1)	13,793 (55.6)	14,084 (55.6)	13,861 (55.0)	< 0.001
Female	44,160 (44.2)	10,588 (42.9)	10,994 (44.4)	11,236 (44.4)	11,342 (45.0)	
Comorbidities						
Hypertension	62,110 (62.1)	16,050 (65.0)	15,632 (63.1)	15,507 (61.2)	14,921 (59.2)	< 0.001
Diabetes mellitus	37,082 (37.1)	9,535 (38.6)	9,390 (37.9)	9,176 (36.2)	8,981 (35.6)	< 0.001
Heart failure	12,213 (12.2)	3,653 (14.8)	3,119 (12.6)	2,812 (11.1)	2,629 (10.4)	< 0.001
COPD	32,832 (32.8)	8,667 (35.1)	8,202 (33.1)	7,984 (31.5)	7,979 (31.7)	< 0.001
ESRD	7,455 (7.5)	2,124 (8.6)	1,894 (7.6)	1,807 (7.1)	1,630 (6.5)	< 0.001
Liver cirrhosis	4,039 (4.0)	1,045 (4.2)	1,066 (4.3)	995 (3.9)	933 (3.7)	0.002
Cerebrovascular accidents	23,637 (23.6)	6,655 (26.9)	5,948 (24.0)	5,708 (22.5)	5,326 (21.1)	< 0.001
Degree of urbanization						
Urban	57,323 (57.3)	13,453 (54.5)	13,475 (54.4)	14,526 (57.4)	15,869 (63.0)	< 0.001
Suburban	31,570 (31.6)	8,079 (32.7)	8,302 (33.5)	8,069 (31.9)	7,120 (28.3)	
Rural	9,999 (10.0)	2,907 (11.8)	2,610 (10.5)	2,552 (10.1)	1,930 (7.7)	
Unknown	1,117 (1.1)	260 (1.1)	400 (1.6)	173 (0.7)	284 (1.1)	
Income level						
Low income	50,762 (50.8)	13,310 (53.9)	12,854 (51.9)	12,808 (50.6)	11,790 (46.8)	< 0.001
Median income	14,869 (14.9)	3,311 (13.4)	3,690 (14.9)	3,751 (14.8)	4,117 (16.3)	
High income	10,183 (10.2)	1,918 (7.8)	2,314 (9.3)	2,687 (10.6)	3,264 (13.0)	
Unknown	24,195 (24.2)	6,160 (24.9)	5,929 (23.9)	6,074 (24.0)	6,032 (23.9)	
Hospital ownership						
Private	68,594 (68.6)	16,219 (65.7)	17,210 (69.4)	20,692 (81.7)	14,473 (57.4)	< 0.001
Public	31,415 (31.4)	8,480 (34.3)	7,577 (30.6)	4,628 (18.3)	10,730 (42.6)	
Hospital region						
North	46,626 (46.6)	10,395 (42.1)	11,165 (45.0)	10,090 (39.8)	14,976 (59.4)	< 0.001
Middle	18,547 (18.5)	4,731 (19.2)	5,142 (20.7)	3,684 (14.5)	4,990 (19.8)	
South	33,062 (33.1)	8,688 (35.2)	7,591 (30.6)	11,546 (45.6)	5,237 (20.8)	
East	1,774 (1.8)	885 (3.6)	889 (3.6)	0 (0.0)	0 (0.0)	
Medical center status						
Non-medical center	46,087 (46.1)	17,336 (70.2)	14,594 (58.9)	11,635 (46.0)	2,522 (10.0)	< 0.001
Medical center	53,922 (53.9)	7,363 (29.8)	10,193 (41.1)	13,685 (54.0)	22,681 (90.0)	
Surgeon age						
< 45	42,564 (42.6)	14,836 (60.1)	14,935 (60.3)	9,836 (38.8)	2,957 (11.7)	< 0.001
≥ 45	57,445 (57.4)	9,863 (39.9)	9,852 (39.7)	15,484 (61.2)	22,246 (88.3)	
Surgeon sex						
Male	98,386 (98.4)	23,937 (96.9)	23,926 (96.5)	25,320 (100.0)	25,203 (100.0)	< 0.001
Female	1,623 (1.6)	762 (3.1)	861 (3.5)	0 (0.0)	0 (0.0)	
Years as a certified surgeon (IQR)	8.6 (5.7–11.9)	6.0 (2.8–9.5)	7.5 (5.0–10.8)	9.4 (6.7–12.3)	10.8 (8.0–13.6)	< 0.001

*IQR* interquartile range, *COPD* chronic obstructive pulmonary disease, *ESRD* end-stage renal disease.

### Comparing different surgical volumes for 5-year death

**Figure 3:**
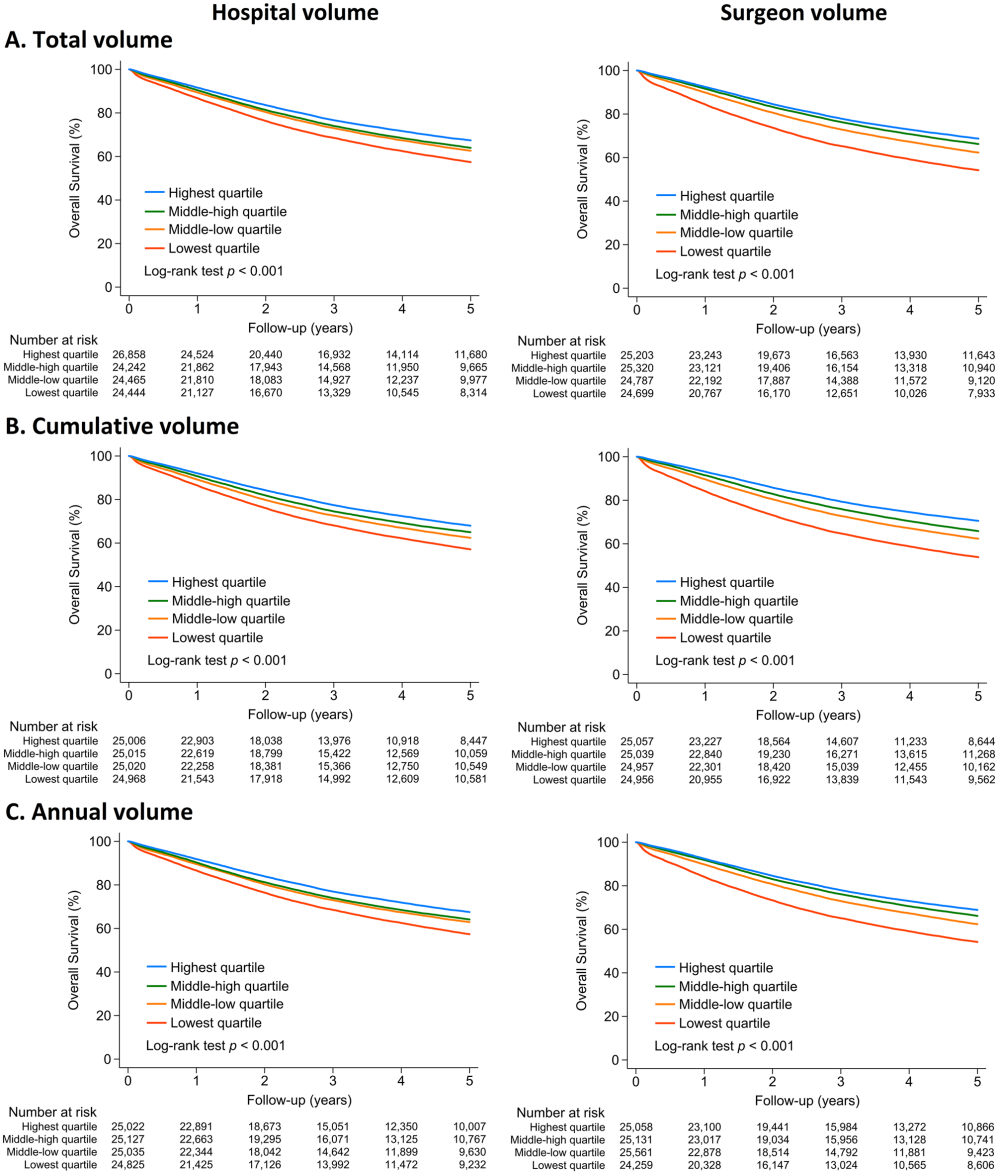
Five-year survival between patients receiving definitive surgery for colorectal cancer divided by different definitions of hospital and surgeon volumes.

We divided CRC patients who received definitive surgeries into quartiles according to different definitions of provider volume. The Kaplan–Meier curves show that patients' 5-year survival was significantly better in other models in higher-volume groups (Fig. 3). The total volume-mortality relationship in CRC surgery is shown in Table 2. Model 1 was adjusted for patient-level risk factors, including age, sex, comorbidities, degree of urbanization, and income group. Model 2 was adjusted for variables listed in Model 1 and hospital-level risk factors, including ownership, geographic region, and medical center status. Model 3 was adjusted for patient-level, hospital-level, and surgeon-level characteristics. The univariate Cox proportional hazards analysis showed that hospital and surgeon total volumes were significantly associated with a lower mortality risk within the 5-year observation period, and after adjustment for individual and provider characteristics, surgeon volume, but not hospital volume, remained a significantly predictive factor of death, with a dose–response relationship. The adjusted HRs were 0.76 (95% CI 0.73–0.78; *p* < 0.001), 0.73 (95% CI 0.70–0.75; *p* < 0.001) and 0.67 (95% CI 0.64–0.70; *p* < 0.001) for the middle-low, middle-high, and highest surgeon total volume group, respectively, compared to the lowest quartile surgeon total volume group.

**Table 2:** Risk factors for 5-year mortality among patients receiving definitive surgery for colorectal cancer.

Variables	Crude HR (95% CI)	*p* value	Model 1		Model 2		Model 3	
			Adjusted HR (95% CI)	*p* value	Adjusted HR (95% CI)	*p* value	Adjusted HR (95% CI)	*p* value
Age ≥ 65	1.75 (1.71–1.79)	< 0.001	1.43 (1.40–1.47)	< 0.001	1.43 (1.39–1.47)	< 0.001	1.43 (1.39–1.47)	< 0.001
Sex (male)	1.14 (1.12–1.17)	< 0.001	1.17 (1.14–1.20)	< 0.001	1.16 (1.14–1.19)	< 0.001	1.17 (1.14–1.19)	< 0.001
Comorbidities								
Hypertension	1.30 (1.27–1.33)	< 0.001	0.93 (0.91–0.96)	< 0.001	0.94 (0.91–0.96)	< 0.001	0.93 (0.91–0.96)	< 0.001
Diabetes mellitus	1.19 (1.16–1.21)	< 0.001	1.01 (0.98–1.03)	0.648	1.01 (0.98–1.03)	0.521	1.01 (0.98–1.03)	0.525
Heart failure	1.72 (1.67–1.77)	< 0.001	1.35 (1.31–1.39)	< 0.001	1.33 (1.29–1.38)	< 0.001	1.34 (1.30–1.38)	< 0.001
COPD	1.31 (1.28–1.34)	< 0.001	1.04 (1.02–1.07)	0.001	1.05 (1.02–1.07)	0.000	1.04 (1.02–1.07)	0.001
ESRD	1.68 (1.62–1.74)	< 0.001	1.32 (1.27–1.37)	< 0.001	1.32 (1.27–1.37)	< 0.001	1.32 (1.27–1.37)	< 0.001
Liver cirrhosis	1.61 (1.54–1.69)	< 0.001	1.45 (1.38–1.52)	< 0.001	1.46 (1.39–1.53)	< 0.001	1.45 (1.38–1.52)	< 0.001
Cerebrovascular accidents	1.55 (1.52–1.59)	< 0.001	1.25 (1.21–1.28)	< 0.001	1.24 (1.20–1.27)	< 0.001	1.24 (1.21–1.27)	< 0.001
Degree of urbanization								
Urban	Reference		Reference		Reference		Reference	
Suburban	1.14 (1.11–1.17)	< 0.001	1.06 (1.03–1.08)	< 0.001	1.05 (1.02–1.08)	0.000	1.05 (1.03–1.08)	< 0.001
Rural	1.32 (1.28–1.37)	< 0.001	1.09 (1.05–1.13)	< 0.001	1.09 (1.05–1.13)	< 0.001	1.09 (1.05–1.13)	< 0.001
Income group								
Low income	Reference		Reference		Reference		Reference	
Median income	0.67 (0.65–0.69)	< 0.001	0.80 (0.77–0.83)	< 0.001	0.81 (0.78–0.84)	< 0.001	0.81 (0.78–0.84)	< 0.001
High income	0.61 (0.58–0.63)	< 0.001	0.72 (0.69–0.75)	< 0.001	0.74 (0.71–0.78)	< 0.001	0.73 (0.70–0.77)	< 0.001
Hospital total volume								
Lowest quartile	Reference		Reference				Reference	
Middle-low quartile	0.83 (0.81–0.86)	< 0.001	0.84 (0.76–0.93)	0.001			0.97 (0.86–1.08)	0.550
Middle-high quartile	0.79 (0.77–0.82)	< 0.001	0.77 (0.66–0.90)	0.001			0.91 (0.76–1.09)	0.302
Highest quartile	0.70 (0.68–0.72)	< 0.001	0.74 (0.60–0.92)	0.008			1.00 (0.78–1.27)	0.966
Hospital ownership								
Private	Reference		Reference				Reference	
Public	1.01 (0.98–1.03)	0.626	0.98 (0.91–1.06)	0.589			0.92 (0.85–1.00)	0.043
Hospital geographic region								
North	Reference		Reference				Reference	
Middle	1.22 (1.19–1.26)	< 0.001	1.10 (1.00–1.21)	0.049			1.12 (1.01–1.24)	0.029
South	1.14 (1.11–1.17)	< 0.001	1.08 (0.99–1.16)	0.078			1.08 (0.99–1.17)	0.099
East	1.42 (1.31–1.53)	< 0.001	1.18 (0.98–1.41)	0.080			1.12 (0.92–1.37)	0.256
Medical center status								
Non-medical center	Reference		Reference				Reference	
Medical center	0.82 (0.80–0.84)	< 0.001	1.00 (0.87–1.15)	0.999			0.99 (0.84–1.16)	0.903
Surgeon total volume								
Lowest quartile	Reference				Reference		Reference	
Middle-low quartile	0.75 (0.72–0.77)	< 0.001			0.60 (0.55–0.67)	< 0.001	0.76 (0.73–0.78)	< 0.001
Middle-high quartile	0.65 (0.63–0.66)	< 0.001			0.57 (0.50–0.66)	< 0.001	0.73 (0.70–0.75)	< 0.001
Highest quartile	0.59 (0.57–0.61)	< 0.001			0.54 (0.44–0.65)	< 0.001	0.67 (0.64–0.70)	< 0.001
Surgeon age								
< 45	Reference				Reference		Reference	
≥ 45	0.82 (0.80–0.84)	< 0.001			0.87 (0.84–0.91)	< 0.001	0.92 (0.89–0.95)	< 0.001
Surgeon sex								
Male	0.89 (0.81–0.96)	0.005			0.88 (0.74–1.05)	0.154	0.96 (0.88–1.06)	0.418
Female	Reference				Reference		Reference	
Experience in surgeon ≥ 5 years	0.75 (0.73–0.77)	< 0.001			0.87 (0.84–0.91)	< 0.001	0.92 (0.89–0.95)	< 0.001

Model 1 was adjusted for patient and hospital characteristics, Model 2 for patient and surgeon characteristics, and Model 3 for characteristics of patients, hospitals, and surgeons. The three models use a Cox model. The surgeon-level random effects were adjusted by using a frailty model for Cox regression.

*HR* hazard ratio, *CI* confidence interval, *COPD* chronic obstructive pulmonary disease, *ESRD* end-stage renal disease.

We further used two definitions of surgical volume that affected the outcomes: cumulative and annual volume. Compared with other definitions of surgical volume, cumulative volume provided the lowest AIC and BIC for predicting 5-year mortality compared with the total and annual volumes. The details are shown in Table 3. For the cumulative volume, the results show that higher surgeon cumulative volume was associated with a lower risk of 5-year mortality than low-volume surgeon volumes. The adjusted HRs for the middle-low, middle-high, and highest quartile compared with the lowest surgeon cumulative volume were 0.75 (95% CI 0.73–0.78; *p* < 0.001), 0.68 (95% CI 0.66–0.71; *p* < 0.001) and 0.59 (95% CI 0.56–0.62; *p* < 0.001), respectively.

**Table 3:** Risk factors for 5-year mortality among patients receiving definitive surgery for colorectal cancer.

Characteristics	Univariate analysis				Multivariate analysis				AIC	BIC
	Hospital volume		Surgeon volume		Hospital volume		Surgeon volume			
	HR (95% CI)	*p* value	HR (95% CI)	*p* value	HR (95% CI)	*p* value	HR (95% CI)	*p* value		
Total volume										
Lowest quartile	Reference		Reference		Reference		Reference		729,662.7	729,906.3
Middle-low quartile	0.83 (0.81–0.86)	< 0.001	0.75 (0.72–0.77)	< 0.001	0.97 (0.86–1.08)	0.550	0.76 (0.73–0.78)	< 0.001		
Middle-high quartile	0.79 (0.77–0.82)	< 0.001	0.65 (0.63–0.66)	< 0.001	0.91 (0.76–1.09)	0.302	0.73 (0.70–0.75)	< 0.001		
Highest quartile	0.70 (0.68–0.72)	< 0.001	0.59 (0.57–0.61)	< 0.001	1.00 (0.78–1.27)	0.966	0.67 (0.64–0.70)	< 0.001		
Cumulative volume										
Lowest quartile	Reference		Reference		Reference		Reference		729,453.3	729,696.8
Middle-low quartile	0.84 (0.81–0.86)	< 0.001	0.74 (0.72–0.76)	< 0.001	0.98 (0.94–1.02)	0.234	0.75 (0.73–0.78)	< 0.001		
Middle-high quartile	0.76 (0.74–0.78)	< 0.001	0.65 (0.63–0.67)	< 0.001	0.91 (0.86–0.96)	0.001	0.68 (0.66–0.71)	< 0.001		
Highest quartile	0.67 (0.65–0.69)	< 0.001	0.54 (0.52–0.56)	< 0.001	0.85 (0.79–0.91)	< 0.001	0.59 (0.56–0.62)	< 0.001		
Annual volume										
Lowest quartile	Reference		Reference		Reference		Reference		729,546.5	729,790.0
Middle-low quartile	0.83 (0.80–0.85)	< 0.001	0.74 (0.72–0.76)	< 0.001	0.97 (0.92–1.02)	0.246	0.76 (0.73–0.78)	< 0.001		
Middle-high quartile	0.79 (0.77–0.81)	< 0.001	0.65 (0.63–0.67)	< 0.001	1.00 (0.93–1.08)	0.946	0.71 (0.68–0.74)	< 0.001		
Highest quartile	0.69 (0.67–0.71)	< 0.001	0.58 (0.57–0.60)	< 0.001	0.99 (0.90–1.09)	0.882	0.66 (0.63–0.69)	< 0.001		

*HR *hazard ratio, *CI* confidence interval, *AIC* Akaike information criterion, *BIC* Bayesian information criterion.

### Sensitivity analysis

The surgeon cumulative volume seems the most suitable for demonstrating the relationship between provider volume and 5-year mortality. The cumulative volume model provided the highest Harrell’s C statistic (0.621), with total and annual volume following. Harrell’s C statistics were 0.618 and 0.620 for total and annual volume, respectively.

### Volumes of laparoscopic minimally invasive surgeries

A total of 17,002 (17.0%) CRC patients who underwent laparoscopic colon resection were enrolled across 118 hospitals and 582 surgeries. Laparoscopy provided a lower 5-year mortality rate among all patients than open surgery (HR 0.60, 95% CI 0.58–0.62; *p* < 0.001). Furthermore, we calculated the number of laparoscopic minimally invasive surgeries for hospitals and surgeries, classifying them into four groups based on their quartiles. Supplementary Fig. 1 illustrates the survival curves for the total volume of minimally invasive surgeries in CRC patients. We observed some differences between the hospital volume and surgeon volume groups, which were statistically significant (all *p* < 0.001). There was an association between 5-year mortality and surgeon volume in the total volume, but not hospital volume. The patients treated by higher-volume surgeons had a lower 5-year mortality rate. The adjusted HRs of 5-year mortality for low-intermediate, high-intermediate, and high-volume compared with low-volume surgeons were 0.87 (95% CI 0.79–0.97; *p* = 0.013), 0.84 (95% CI 0.75–0.95; *p* = 0.007) and 0.90 (95% CI 0.79–1.04; *p* = 0.159), respectively. However, regardless of hospital and surgeon volume, using different definitions of volume to include cumulative and annual volumes was not associated with 5-year mortality (Supplemental Table 1).

## Discussion

The results of our study reveal that higher surgeon volume is independently associated with the lower 5-year mortality rate in CRC patients receiving definitive surgery after adjusting for hospital volume and other potential confounders. When introducing different definitions of provider volume, the inverse association between surgeon volume and 5-year mortality rate remain consistently significant, while hospital volume does not. Surgeon volume is more important than hospital volume in CRC surgeries. Thus, those CRC patients treated by high-volume surgeons at low-volume hospitals have a lower mortality risk than those treated by low-volume surgeons at high-volume hospitals. In addition, our findings demonstrate that “cumulative volume” can predict the 5-year mortality of the study cohort better than total and annual volume.

There is a large body of literature over the past 30 years investigating the volume-outcome relationship in cancer treatment^26^. However, the definition of provider volume used in these studies varies and therefore a different definition of provider volume could affect the analytical results of patient outcomes. The most common volume definitions are annual volume and cumulative volume^17,27–30^. In a volume-outcome study on cervical cancer patients after radiation therapy conducted by Wright et al., mean annualized hospital volume and previous year hospital volume were not associated with survival benefit, while in the sensitivity analysis, current year hospital volume, which was defined as the number of patients treated at a given hospital within the same calendar year, significantly predicted survival outcome^19^. Derogar et al. reported annual volume as the number of operations within the index year and cumulative volume as the chronological number of operations, and they found the combination of annual and cumulative surgeon volume to be a predictor of long-term survival for esophageal cancer patients, whereas individually these factors were not^17^. Jeldres et al. reported that both annual and cumulative provider volumes were independent predictors of failure-free survival in patients with localized prostate cancer after definitive radiotherapy^18^. In the present study, we explored the effect of varying definitions of provider volume on the mortality rate of CRC patients, and the results were consistent among different models. Moreover, we also include AIC, BIC, and Harrell’s C statistics to examine the fitness of varying volume definitions, which was not included in the prior studies. When examining AIC, BIC, and Harrell’s C statistics with different volume definitions, cumulative volume, defined as the number of surgeries the surgeon performs before the index surgery, is the best-fitting model. This number could present as an indicator of the cumulative experiences of surgery. In addition, this finding can also be used as a guide in subspecialty education programs, showing that the cumulative procedures conducted by the surgeon should meet the minimal requirement to be certified as a specialist.

The surgeon's cumulative volume could be seen as a measure of accumulated experience and proficiency. Surgeons who have performed more surgeries are expected to have better skills and outcomes due to their experience. Notably, some medical procedures have a learning curve, with surgeries likely improving over time with practice. In this context, the cumulative volume could assess how the performance of surgery changes as surgeons gain more experience, aligning with the “practice makes perfect” hypothesis^26,31^. Moreover, cumulative volume might be considered a proxy for the quality of care. Surgeries performed by higher-cumulative-volume surgeons are thought to be more efficient and effective as potentially accruing more experience leads to improved skills, reduced complications, and enhanced patient care.

The positive volume-outcome relationship in CRC patients after surgery has been demonstrated in much of the literature, with or without mutual adjustment for hospital and physician volume. Schrag et al*.* investigated hospital and surgeon volume on the outcome of rectal cancer patients. The results of their work revealed that surgeon volume was a better predictor of long-term survival than hospital volume^7^. In the results from a Cochrane systematic review and meta-analysis, the effect of workload on patients’ prognosis after CRC surgery was stronger at the surgeon level than at the hospital level, particularly in the outcome of 5-year overall survival and operative mortality^32^. Previous studies have explored the positive association of high cumulative and high annual volume surgeons on cancer survival rates for rectal and colorectal cancer patients^24,30^. When researching different volume definitions, the results of our study confirmed the robustness of the importance of physician volume over hospital volume on the long-term mortality rate in CRC patients after cancer surgery. These findings suggested that enhancing surgeon-specific experiences can improve patients’ outcomes. Thus, from a healthcare policy standpoint, minimal requirements for surgical procedures and specialization may be essential for surgeon training in colorectal cancer surgery. In Taiwan, patients can seek second opinions with great accessibility under the coverage of National Health Insurance. As a result, patients tend to visit medical centers for cancer treatment. From the patients’ aspect, the information on surgeon volume can minimize the information gap and help patients choose an experienced surgeon in the local hospital near where they live, thus improving patient logistics of high-volume centers.

The laparoscopic surgical approach has become the gold standard treatment for most colon cancers, as recommended by the American Society of Colon and Rectal Surgeons^33^. Our study found no association between the volume of laparoscopic minimally invasive surgeries and the risk of 5-year mortality due to the smaller number of patients included in the analyses during the later years of follow-up. The adoption of the laparoscopic approach for colorectal surgery within the surgical community has been slower and less widespread in Taiwan compared to laparoscopic procedures for other indications. In our study population, we observed a gradual increase in the number of patients with CRC selected for laparoscopy in recent years. The implementation of laparoscopic surgery, like any new surgical technique, is associated with a learning curve and may result in longer operation times, shorter hospital stays, reduced incidence of surgical site infection, increased complications, or a higher frequency of conversion to open surgery^34–37^.

The current study had certain limitations. First, several potential confounders, including smoking, alcohol consumption, obesity, and family history, are not available in the data. Secondly, we lacked data on tumor markers and genetic features, which might also impact patients’ long-term survival. Third, potentially curative surgeries might be mixed with palliative operations. Fourth, surgical volume beyond the study period was not collected. Finally, a more extended surgical experience may be an effect modifier for surgeon volume, which needs further studies.

In this nationwide population-based study, we observed that CRC patients treated by high-caseload surgeons had better survival. Additionally, surgeon volume is shown to be a more important predictor of 5-year mortality than hospital volume in CRC patients after cancer surgery. Among the varying definitions of surgeon volume we applied, surgeon cumulative volume could predict patients’ outcomes better than the volumes of the other definitions. Hence, the cumulative volume of the surgeon can serve as an index of surgeon experience and be used as a reference in subspecialty qualification.

### Supplementary Information

Supplementary Information.

## Data Availability

The datasets used and analyzed during the current study are available from the corresponding author upon reasonable request.
