# Supplementary material for: The impact of surgical volume on outcomes in newly diagnosed colorectal cancer patients receiving definitive surgeries
Source: Sci Rep. 2024 Apr 8;14:8227. doi: 10.1038/s41598-024-55959-w (PMC11001606; doi:10.1038/s41598-024-55959-w)
Supplement: Supplementary file 1 — Supplementary Information. [file 41598_2024_55959_MOESM1_ESM.docx]

**Supplementary Material**

**Supplementary Table 1** Impact of the volume of minimally invasive surgeries and five-year mortality in colorectal cancer patients

| **The volume of minimally**  **invasive surgeries** | | **Univariate analysis** | | | |  | **Multivariate analysis** | | | |  | |  |
| --- | --- | --- | --- | --- | --- | --- | --- | --- | --- | --- | --- | --- | --- |
|  |  | **Hospital volume** | | **Surgeon volume** | |  | **Hospital volume** | | **Surgeon volume** | |  |  |  |
|  |  | **HR (95% CI)** | ***p* value** | **HR (95% CI)** | ***p* value** |  | **HR (95% CI)** | ***p* value** | **HR (95% CI)** | ***p* value** | **AIC** | **BIC** | |
| Total volume | Lowest quartile | reference |  | reference |  |  | reference |  | reference |  | 58,800.2 | 58,995.1 | |
|  | Middle-low quartile | 0.99 (0.81–1.20) | 0.904 | 0.79 (0.67–0.92) | 0.003 |  | 1.03 (0.87–1.22) | 0.744 | 0.87 (0.79–0.97) | 0.013 |  |  | |
|  | Middle-high quartile | 0.79 (0.61–1.03) | 0.078 | 0.74 (0.61–0.89) | 0.002 |  | 1.05 (0.84–1.31) | 0.659 | 0.84 (0.75–0.95) | 0.007 |  |  | |
|  | Highest quartile | 0.87 (0.61–1.25) | 0.465 | 0.80 (0.62–1.03) | 0.082 |  | 1.14 (0.86–1.53) | 0.364 | 0.90 (0.79–1.04) | 0.159 |  |  | |
| Cumulative volume | Lowest quartile | reference |  | reference |  |  | reference |  | reference |  | 58,807.0 | 59,001.8 | |
|  | Middle-low quartile | 1.05 (0.95–1.15) | 0.369 | 0.92 (0.84–1.02) | 0.109 |  | 1.06 (0.95–1.18) | 0.322 | 0.91 (0.83–1.01) | 0.066 |  |  | |
|  | Middle-high quartile | 0.91 (0.81–1.02) | 0.099 | 0.90 (0.80–1.00) | 0.050 |  | 0.96 (0.84–1.10) | 0.579 | 0.91 (0.80–1.03) | 0.121 |  |  | |
|  | Highest quartile | 0.98 (0.86–1.13) | 0.816 | 0.86 (0.75–0.98) | 0.020 |  | 1.01 (0.86–1.20) | 0.894 | 0.91 (0.78–1.05) | 0.185 |  |  | |
| Annual volume | Lowest quartile | reference |  | reference |  |  | reference |  | reference |  | 58,806.3 | 59,001.1 | |
|  | Middle-low quartile | 1.01 (0.91–1.13) | 0.825 | 0.99 (0.88–1.10) | 0.781 |  | 1.07 (0.96–1.20) | 0.232 | 0.93 (0.84–1.03) | 0.177 |  |  | |
|  | Middle-high quartile | 0.97 (0.85–1.10) | 0.605 | 0.88 (0.78–0.99) | 0.039 |  | 1.04 (0.90–1.20) | 0.590 | 0.90 (0.80–1.00) | 0.057 |  |  | |
|  | Highest quartile | 0.96 (0.82–1.13) | 0.641 | 0.88 (0.76–1.02) | 0.083 |  | 1.04 (0.87–1.24) | 0.650 | 0.88 (0.77–1.01) | 0.071 |  |  | |

HR, hazard ratio; CI, confidence interval; AIC, Akaike information criterion; BIC, Bayesian information criterion


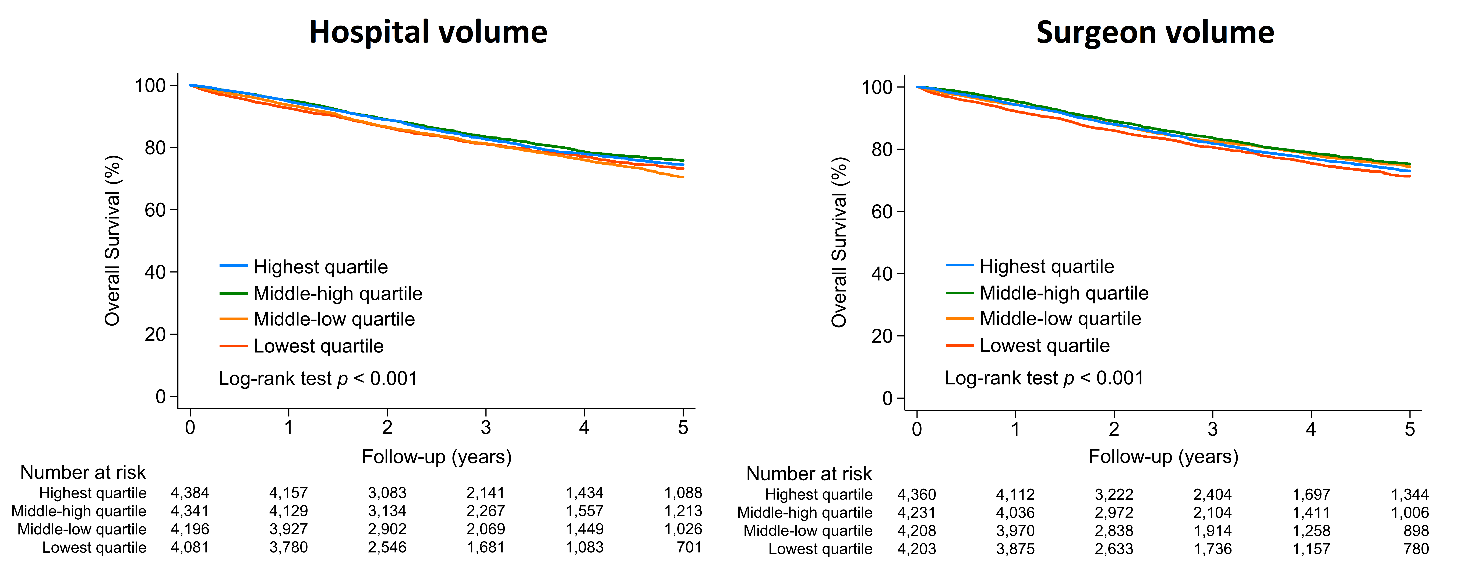


**Supplementary Figure 1** Survival curves for the total volume of minimally invasive surgeries in colorectal cancer patients.
